# A Comprehensive Comparison and Validation of Published Methods to Detect Turn Switch during Alpine Skiing

**DOI:** 10.3390/s21072573

**Published:** 2021-04-06

**Authors:** Aaron Martínez, Cory Snyder, Stephanie R. Moore, Thomas Stöggl

**Affiliations:** 1 Department of Sport and Exercise Science, University of Salzburg, Schlossallee 49, 5400 Hallein/Rif, Austria; cory.snyder@sbg.ac.at (C.S.); stephanie.moore@sbg.ac.at (S.R.M.); thomas.stoeggl@sbg.ac.at (T.S.); 2 Athlete Performance Center, Red Bull Sports, Brunnbachweg 71, 5303 Thalgau, Austria

**Keywords:** accuracy, force plate, IMU, motion capture, pressure insole, precision, sensor, ski

## Abstract

The instant of turn switch (TS) in alpine skiing has been assessed with a variety of sensors and TS concepts. Despite many published methodologies, it is unclear which is best or how comparable they are. This study aimed to facilitate the process of choosing a TS method by evaluating the accuracy and precision of the methodologies previously used in literature and to assess the influence of the sensor type. Optoelectronic motion capture, inertial measurement units, pressure insoles, portable force plates, and electromyography signals were recorded during indoor treadmill skiing. All TS methodologies were replicated as stated in their respective publications. The method proposed by Supej assessed with optoelectronic motion capture was used as a comparison reference. TS time differences between the reference and each methodology were used to assess accuracy and precision. All the methods analyzed showed an accuracy within 0.25 s, and ten of them within 0.05 s. The precision ranged from ~0.10 s to ~0.60 s. The TS methodologies with the best performance (accuracy and precision) were Klous Video, Spörri PI (pressure insoles), Martinez CTD (connected boot), and Yamagiwa IMU (inertial measurement unit). In the future, the specific TS methodology should be chosen with respect to sensor selection, performance, and intended purpose.

## 1. Introduction

Turns are a basic component of alpine skiing performance that have unique features due to the constraints of sport-specific equipment. The specific characteristics of each turn (initial speed, edge angle, centripetal acceleration, etc.) define the outcome of a ski run [1]. To properly assess the magnitude and relevance of the aforementioned characteristics, it is imperative to accurately define the moment when a turn begins and ends; this moment is called the turn switch (TS) point. TS detection has been a well-researched topic for over 30 years in a wide range of fields [2,3,4,5]. Each field, from scientific research, to coaching, to app development, has conceptualized events and implemented methodologies to segment a ski run into ski turns. Despite this, there is little agreement between disciplines as to which methods to detect TS should be used.

A wide variety of signals and observations have been used as the base to develop several methodological approaches for TS detection. The development of technology has constantly provided more options, in the form of new sensors and better signals, to build upon the earlier approaches based on theoretical [2] and observational [6,7] models. From these, two main sensor groups used for TS detection can be identified when analyzing the literature. The first group consists of sensors that provide kinematic data and therefore define TS by evaluating motion. Alternatively, kinetic devices that enable the identification of TS through the analysis of the skier-equipment interaction constitute the second group.

Among the kinematic group, analysis of two-dimensional video to define ski edge change as the TS point has been commonly used in research [8,9] as well as by ski instructors. Nevertheless, it requires a manual selection of the frame when TS occurs, and is dependent on the camera placement to provide a clear image. Motion capture systems (MCS) by means of tracking three-dimensional (3D) trajectories using different video [1,3,4,8,10] or infrared camera systems [11,12] have also been prevalent kinematic measuring devices used. The execution of this data collection during skiing presents several challenges; including the difficulty of accomplishing ideal camera placements on ski terrain, limited capture volumes and subsequently a low number of turns that can be recorded, as well as time-consuming post-processing [1,13]. Despite the methodological challenge, Supej and colleagues [3] used a system of six camcorders to develop a method that defined TS as “the point of intersection between the trajectory of the center of mass (CoM) projection on the snow, and the arithmetic mean of the ski positions”. This concept has been adopted by several research groups and has been shown to be versatile to ski style and skill [1,4,10,14,15,16,17,18,19,20,21,22]. Although 3D motion capture is the gold standard for kinematic data collection, few TS detection methods have been based on these data due to the aforementioned limitations [3,8].

The development of inertial measurement units (IMUs) has increased the flexibility of field data collection compared to that of 3D motion capture. Generally, IMUs do not require extensive preparation and allow for continuous signal recording without limited capture volume. Fasel and colleagues [12] validated an IMU configuration to estimate joint center positions and CoM kinematics during skiing. To accomplish this, they installed an infrared camera system surrounding a ski treadmill, which allowed for the tracking of a large number of turns using a gold standard measurement device (MCS) for kinematic data. Consequently, it provided an excellent context to evaluate the performance of the IMU signals and configuration over a large number of consecutive turns [23]. More recent studies have used this configuration and added position data (GNSS or similar) [24] to assess the skier movement on snow [25,26]. Such data processes have allowed for the reproduction of the aforementioned Supej [3] method on snow, and encouraged the development of methods based on the distance between the CoM and the ankles [13,15]. However, the use of IMUs is not limited to modeling the movement of the whole body during skiing. For instance, events based on the lateral acceleration of the trunk [27], the inclination of the pelvis [28], or the angular velocity of the boot [9] have also been used to define TS. Position sensors (GNSS) used singularly can also approximate TS points using the head or the calculated CoM trajectories as defining signals [29,30,31]. Despite the many published kinematic methodologies, it is not comprehensively clear which is best or how comparable they are.

The combination of kinematic data and muscular activity have also been used to identify TS in the field. Kröll and colleagues [32] defined “a combination of knee angle and raw electromyographic (EMG) data to determine the start and end of each double turn”. More specifically, they identified a pronounced amplitude decrease of the vastus lateralis activity within a zone of interest identified by knee angles. This method is advantageous for segmenting signals when investigating EMG-based research questions, because there is no between-system synchronization needed. However, the collection of EMG data is traditionally a laboratory-based method that requires time-consuming preparations, manual post-processing, and additional care when used in the field.

The kinetic group is composed of two types of sensors: pressure insoles (PI) and portable force plates (PFP; generally located between the bindings and the skis). Both sensors measure the interaction between the skier and the skis. Although force plates are the gold standard sensor for kinetic measurements, when employed during skiing, the devices used in previous studies increase the ski-binding complex weight and may affect the skier’s technique and comfort [33]. PIs are much less invasive and reportedly, do not affect the user’s comfort [28,34]. The main limitation when using PIs is that due to their placement, they can only measure the pressure between the plantar surface of the foot and the boot, instead of all the force transmitted from the skier to the skis (e.g., through the lateral/dorsal surfaces of the foot, or through the cuff of the boot) [35]. Regardless of their inherent differences, the same principles to define TSs are used with both methodologies; the beginning of the turn has been defined as the point of minimum vertical load during a turn [34,36], as the point when the force equals a pre-defined threshold [37], or as the point where the load in both skis is equal [5].

It is clear that there are a number of different sensors and approaches used to assess TS points. However, the information about the comparative performance of the different methods is very limited. Further, most of these methods have primarily been used with a specific turn size. In order to compare and evaluate the performance of the different methodologies, a reference TS methodology is needed. Therefore, the concept the current authors selected to represent a reference TS was that of Supej and colleagues [3]. Their concept is commonly used in alpine ski research to define TS and has been shown to be independent of the skiing level and style [1,10,14,15,16,17,18,19,20,21,22,37]. Accordingly, the TS point reference values used in this manuscript were assessed by applying Supej’s concept to the gold standard trajectory tracking device (MCS), thus ensuring the accuracy of the reference values. Ultimately, to facilitate the process of choosing an adequate TS detection method for alpine skiing, this study aimed to evaluate the accuracy and precision of the TS time points detected with the methodologies previously used in literature and to present a comprehensive timeline of the difference of each TS point method compared to the pre-defined TS reference. Furthermore, the secondary goal was to reproduce the published methods with the original type of sensor and additionally with gold standard sensors to compare the performance between sensor configurations.

## 2. Materials and Methods

### 2.1. Participants and Experimental Approach

Fourteen skiers volunteered to participate in this study. Eleven participants (age: 13.6 ± 0.3 years, height: 1.59 ± 0.08 m, mass: 48.1 ± 7.4 kg) were junior ski-racing athletes at a skiing specific middle school in Austria. Three participants (age: 31.4 ± 9.6 years, height: 1.86 ± 0.01 m, mass: 85.7 ± 1.5 kg) were experienced ski racers. All participants were familiar with skiing on the ski treadmill. Before the measurements were conducted, participants and their guardians were informed of the experimental protocol and potential risks and benefits of the investigation prior to providing written consent. This study was approved by the local ethical committee (EK-GZ: 11/2018), and was conducted in accordance with the Declaration of Helsinki.

**Figure 1:**
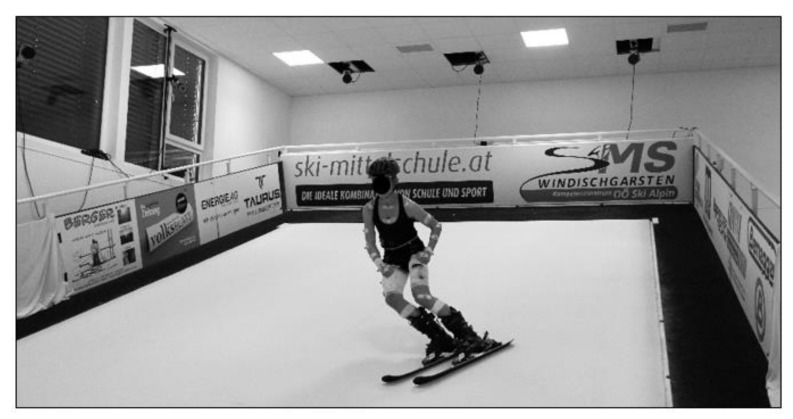
Illustration of the experiment configuration in the indoor skiing carpet.

For the reproduction and comparison of the different turn detection methodologies, an experiment was designed where participants performed ski turns on an indoor ski treadmill (BIG Maxx ISB200, MaxxTracks; see Figure 1). The moving belt of the treadmill, with a skiable area of 9.5 m × 5.0 m, allowed for the execution of successive turns while collecting data from both wearable and stationary sensors during skiing. After instrumentation, each participant performed a standardized warm-up guided by a professional coach, followed by two 30 s trials performed with different turn sizes: long and short. For the long turns, participants were instructed to implement the turns that maximized the treadmill width. Alternatively, the short turns were performed using only the middle 50% of the treadmill width (2.5 m), which were marked with two vertical poles in front of the treadmill within the participants’ field of view. Both conditions were performed at 17° of slope angle and a belt speed of 4.6 m·s^−1^. These conditions represent the most familiar settings used during training on the ski treadmill for the participants in the current study.

### 2.2. Instruments

#### 2.2.1. 3D Motion Capture

A thirteen infrared camera motion capture system (MCS; 11 Oqus 700+, 2 Oqus 500+ Qualysis AB, Göteborg, Sweden) was installed surrounding the ski carpet (see Figure 1). One of the cameras was placed directly in front of the ski treadmill and used for 2D video recording. A marker set of 49 passive-reflective markers (diameter = 14 mm) was placed on body landmarks (modified Helen Hayes [38]) and used to obtain a full body model including joint centers and segment orientations [39]. The markers were placed directly on the skin with exception of the shank, foot and head markers, which were placed on the boot and helmet, respectively. During the static calibration of the system, the skiers were wearing the boots un-buckled to allow for an upright neutral position. The marker trajectories were collected at 100 Hz and filtered with a low-pass, fourth-order, zero-lag Butterworth filter with a cutoff frequency of 6 Hz. This cutoff was selected in order to retain 95% of the marker trajectory signal power [40].

Due to the indoor nature of the data collection, the use of position data sensors was not possible. Instead, position data collected with the 3D motion capture system was used. In order to replicate the methodologies that required posterior head position data (typical placement of position tracking sensors [31]), the head trajectory was defined as the arithmetic mean of the right and left back head marker trajectories (Visual3D v6.03.6 C-Motion, Germantown, MD, USA). Further, the trajectory of the estimated CoM was used [39] to reproduce the methods approximating CoM from the head trajectory measured with position data sensors [15,30].

#### 2.2.2. Inertial Measurement Units

Seven IMUs (Physilog 5, GaitUp, Lausanne, Switzerland) were necessary to recreate the IMU-based TS methods. Six of them were secured to the participants at the shanks, thighs, sacrum, and sternum [12], and one used as a trigger to help partition the measurement into areas of interest (calibration movements, trials, etc.). Signals from the tri-axial accelerometer (range ± 16 g) and the triaxial gyroscope (range ± 2000 deg·s^−1^) were collected at 512 Hz. A calibration routine was performed prior to the participants’ warm up as described by Fasel et al. [12].

The processing of the signals was subsequently adapted to replicate the different TS methods using IMUs. For this, two main groups of methods that used IMUs were identified: methods that used multiple IMUs and those that used single IMUs. The methods based on single IMUs [5,28,40] were processed by replicating filter cutoff frequencies and drift correction approaches individually for each method. The methods which required several IMUs [8,15,16,32], were processed using a validated open source tool [41]. This tool requires the aforementioned calibration routine and calculates rotation matrices to locate and orient the sensors and joint centers in space at each time point [12,24]. Furthermore, it corrects the drift in the signal and calculates knee joint angles [23]. A custom body model (Matlab R2019a, MathWorks, Natick, MA, USA) was then built using previously defined segments [24,39].

#### 2.2.3. Instrumented Boots

All participants completed the trials using ski boots instrumented with an IMU [9] (LSM6DS3, 2.5 × 3 × 0.83 mm, ± 16 g and ± 1000 deg·s^−1^ full scale, STMicroelectonics, Geneva, Switzerland). The IMUs were placed in the back of the upper cuff of each boot, and were fixed using a tight elastic strap and a customized rigid housing to avoid movement. Signals were collected at 833 Hz, analog and digital low-pass filters were applied directly after A/D conversion, and the signal was forwarded via Bluetooth at 54 Hz, thus becoming the final sampling rate. The sensor data was collected and stored by an in-house smartphone application (see detailed description in [9]). These boot IMUs comprised a previously validated system for edge angle estimation [42] and for turn segmentation [9,43]. Therefore, they were not part of the seven IMU configuration mentioned in the Section 2.2.2. In the current study, a new TS detection method was included using the gyroscope signal (Martinez Yaw, see Table 1). It is based on the hypothesis that TS occurs when the averaged yaw angular velocity of both boots equals zero. After resampling, signals were filtered for each method accordingly (for Martinez Yaw a 6 Hz low pass Butterworth filter was used).

#### 2.2.4. Pressure Insoles

A pair of wireless PIs (Loadsol^TM^, Novel GmbH, Munich, Germany) were placed inside the ski boot after selecting the proper size for each skier. The insoles consisted of two sensor-regions that separated the foot area into fore and aft sections. Manufacturer calibration routines were performed prior the data collection. Signals were collected at its maximum sampling rate (100 Hz) and filtered with a low pass 6 Hz Butterworth filter [3,9,34]. The fore/aft sensor-regions of each PI were combined for all the subsequent calculations.

#### 2.2.5. Force Plates

The PFP system was integrated by four dynamometers (Kistler GmbH, Winterthur, Switzerland) mounted under the toe and heel binding of each ski [33]. The dynamometers weighed 0.9 kg each and were 36 mm in height. Amplifiers, data loggers, power supply, and supply box (4 kg) were carried in a backpack worn by the skiers (see detailed description in [34]). The magnitude of the combined weight limited the number of skiers able to use this system (*n* = 2). Signals were collected at 200 Hz (maximum sampling rate), resampled to 100 Hz (in order to match the sampling rate of the MCS) and filtered with a 6 Hz low pass Butterworth filter [5,36].

#### 2.2.6. Electromyography

Muscle activity (EMG) was collected from the *vastus lateralis* muscle unilaterally on the right leg [32]. After shaving and cleaning the skin with isopropyl wipes, a bipolar surface electrode (Ag/AgCl HEX Dual, Noraxon Inc, Scottsdale, AZ, USA) was placed following the SENIAM recommendations [44]. The electrodes had a diameter of 10 mm and an inter-electrode distance of 20 mm. The signal was amplified and band-pass filtered at the source (5–500 Hz, 100 dB, Ultium^®^ EMG Sensor System, Noraxon, Scottsdale, AZ, USA) and recorded at 2000 Hz (Ultium receiver, Noraxon, integrated on Qualisys Track Manager 2019.3, Qualisys, Gothenburg, Sweden).

### 2.3. Turn Detection Methodologies

The summary and details of all TS detection methodologies assessed in this study can be found in Table 1. All TS detection methods were replicated following the procedures stated in their respective references. When the authors were not able to interpret or reproduce a method, the publication’s authors were contacted for clarification in order to properly replicate the original method. After inspection of the signal quality, data from specific participants were removed from the analysis of certain methods due to technical failures. The subsequent number of participants used for each method is reported in Table 1. Some TS detection methods were developed using wearable sensors but have not yet been compared to a gold standard data acquisition device. For such methods, data was collected using two systems: the original one used in literature and the corresponding gold standard data acquisition device. Subsequently, some methods appear twice in the results, initially assessing the performance as previously published (e.g., with wearable sensors), and secondly assessing the performance with a gold standard device (therefore evaluating the concept rather than the measuring system) [1,5,8,15,16,40].

### 2.4. Data Analysis

The synchronization between sensors was performed by means of a movement-based synchronization routine before and after every trial. The routine consisted of lifting the right leg for three seconds, followed by an intense stomp and a three second pause with the leg unloaded. This stomp event was detected with IMUs, instrumented boot, PI, PFP, and MCS, thus enabling the synchronization between all systems. The EMG signal was collected using the MCS software and therefore was already digitally synchronized. All the signals, independent of their sampling rate, were resampled using Piecewise Cubic Hermite Interpolating Polynomial (PCHIP [45]). The resampling was performed between the synchronization stomps using the 3D motion capture signal length as the reference length.

**Figure 2:**
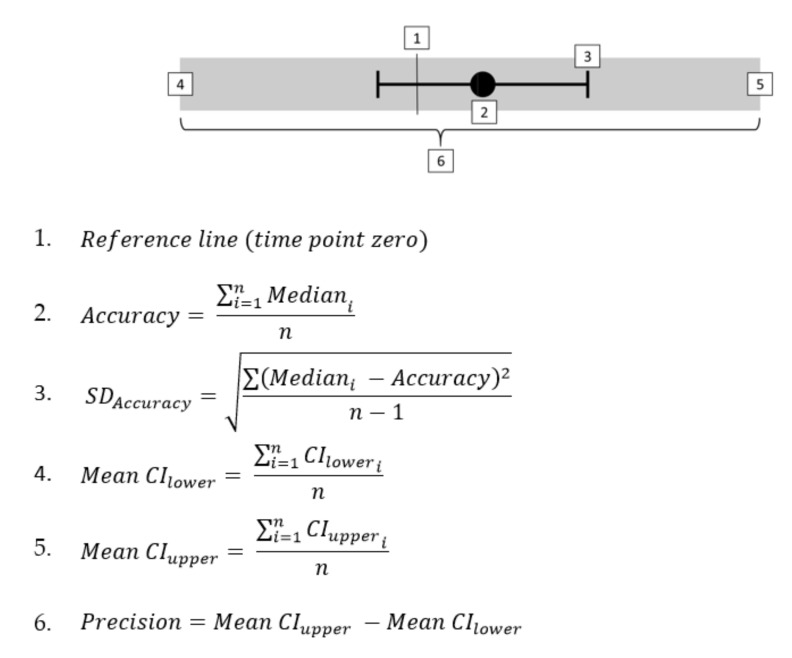
Graphical illustration and equations to clarify the interpretation of the data presented in the results timeline. *i*, participant index; n, number of participants per method; *SD* , standard deviation; CI_lower_, lower confidence interval limit (percentile 2.5); CI_upper_, upper confidence interval limit (percentile 97.5).

Time differences between each methodology and the reference were used to assess the accuracy and precision. The calculation of these time difference was performed by subtracting the reference TS time point of every turn from the associated TS in each methodology. Normality of the time differences for each method was assessed by means of a Shapiro–Wilk test. Due to the non-normal distribution of several methods analyzed, non-parametric procedures were used to describe the data. Therefore, the median and the lower and upper confidence interval (CI) limits (percentiles 2.5 and 97.5 respectively) were calculated per participant, method, and turn size. The medians and CIs of all participants were then pooled into a new data set for each method and turn size. Subsequently, the mean and standard deviation of all participant medians were used to evaluate the accuracy of each method (see equations 2 and 3 in Figure 2). The precision was calculated as the range between the mean upper and lower CI across all participants per method (see equations 4–6 in Figure 2) [24,46].

Wilcoxon–Mann–Whitney tests were used to evaluate pairwise differences between time points measured with two different sensors assessing the same concept (α = 0.05). These comparisons were performed by pooling together all turns, participants, and turn sizes for both methods involved in comparison. Each TS method that was evaluated with two sensors can be seen in Table 1. Further comparisons of central-tendency between methods were deemed impractical due to the alpha corrections that would be necessary for the vast number of comparisons possible.

Finally, the aforementioned accuracy and precision metrics were used to define a timeline to depict each method against the reference (represented as time point zero). The methods were ordered from those that detected TS before the reference (negative mean differences) to those that detected TS after the reference (positive mean differences). The mean upper and lower confidence intervals were then added to depict precision graphically (Figure 2).

## 3. Results

**Figure 3:**
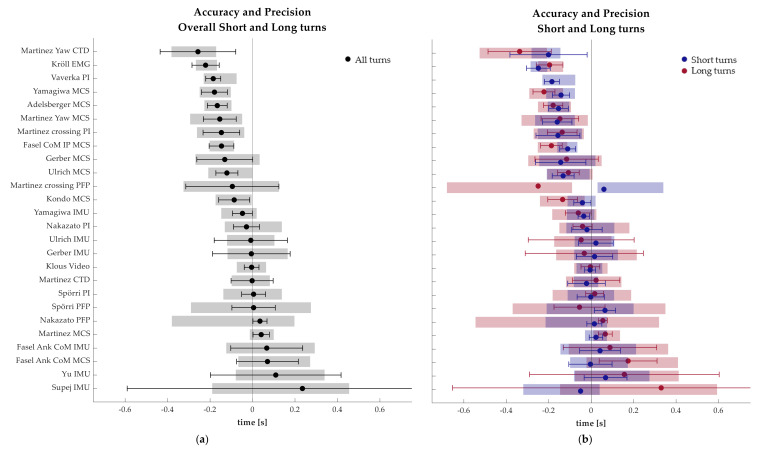
Timeline depicting the accuracy and precision of the turn switch detection methodology. The left side (**a**) figure shows the accuracy (black dot), standard deviation of the accuracy (whiskers) and precision (shaded area) of every method calculated with all turns pooled together. The right side (**b**) graph separates the data by turn size, long turns (red) and short turns (blue).

The timeline graphically depicting the accuracy and precision results can be found in Figure 3. The results (presented in Figure 3) show the distribution of methods from earliest to latest in relation to the reference. The left side figure includes all turns, and the right side figure differentiates between long (red) and short (blue) turns. The timeline with all turns shows ten methodologies with an accuracy better than 0.05 s. However, the standard deviations of their accuracy show different levels of variability, ranging from less than ±0.04 s for Nakazato PFP, Klous Video and Martinez MCS to almost ±0.2 s for Ulrich IMU and Gerber IMU. From the remaining methodologies, 12 tended to identify the TS before the actual event, and four of them detected them with some delay. Among the ten most accurate methodologies, the most precise were Martinez MCS, Klous Video, Martinez CTD and Yamagiwa IMU with a precision better than 0.2 s. Nevertheless, Kröll EMG, Fasel CoM IP MCS, Adelsberger MCS, Vaverka PI and Kondo MCS also had a precision under 0.2 s.

The differences between sensors assessing the same TS concept are presented in Table 2. All methods but two (Nakazato [34] and Spörri [36]) show statistically significant differences between the two sensor groups compared. However, the turns included in these two methods were collected from just two skiers due to the weight of the necessary equipment (see Section 2.2.5). The absolute time difference between sensors yielded values ranging from 0.00 s (Spörri) to 0.18 s (Gerber).

## 4. Discussion

The main purpose of the current study was to create a timeline that would facilitate the comparison of accuracy and precision between the different TS detection methodologies used in literature. All the methods analyzed show an accuracy within ± 0.25 s from the reference, with ten of them having an accuracy better than 0.05 s. The precision ranges from ~0.10 s to ~0.60 s. The TS methodologies with the best combined accuracy and precision are Klous Video [8], Spörri PI [36], Martinez MCS and CTD [9], and Yamagiwa IMU [27].

Four of the TS methods were directly based on the participants’ trajectories (Supej MCS [3], Kondo MCS [31], Adelsberger MCS [30], and Fasel CoM IP MCS [15]). Although the reference method Supej MCS [3] was based on participant trajectories, it cannot be compared or evaluated because it is implicit in the values of all other methods (i.e., TS = 0). The three remaining methods were based on position data collected with GNSS or similar sensors. In this study, trajectories assessed with MCS were utilized instead (due to the indoor nature of the data collection), thus ensuring the accuracy of the data. Kondo MCS [31] was based on the head trajectory and revealed the best results among the trajectory based methods with an accuracy of −0.04 s and a precision of 0.13 s for short turns. Alternatively, Adelsberger [30] and Fasel [15] used an approximation of the CoM based on the head trajectory, though in this manuscript the CoM trajectory defined by MCS was used. Although the approximation was validated (accuracy was reported as 0.08 m; [24]), the direct use of the MCS CoM trajectory likely improves the results of the assessed methodologies because it eliminates any error associated with the CoM trajectory approximation. Despite eliminating potential errors in trajectory estimation, the results indicate that TS methodologies purely based on head or CoM trajectories tend to estimate the instant of TS early. One possible explanation for this offset may be rooted in the skiers’ technique: upper body compensations may exist to aid the skiers’ weight transition, which will ultimately influence the CoM and head trajectories. These three methods performed better for the more dynamic short turns rather than the long turns as can be seen in Figure 3. Fasel and colleagues [15] also reported values for accuracy and precision (against MCS) when proposing their method. Although the reported accuracy (+0.008 s) and precision (±0.044 s) are excellent, the system’s sampling rate was 50 Hz. Nevertheless, the extrapolation of their results to the minimum detectable time unit (0.02 s) would still suggest a very good performance.

When considering that their data was collected from on-snow skiing, it agrees with our results and indicates that the methods based on position data trajectories may perform better when applied to highly dynamic movements (i.e., without treadmill constraints).

The detection of TS based on edge change selected from 2D video data showed the best overall results (Klous Video [8]). The accuracy was within 0.01 s from the reference and the precision was 0.14 s. Although these results highlight the accuracy of the method, there are some practical factors to consider. In this particular data collection, the camera was placed directly in front of the skier, allowing for an unencumbered view of the skier movement in every turn. This is generally not plausible on ski pistes, where a camera would need to be positioned stationary exactly in the frontal plane of the skier during TS, therefore limiting the possible number of turns recorded. Alternatively, a camera could be positioned on the skier or following the skier, in which case distorting factors such as limited perspective, altered distance, additional movement, or vibrations would make the frame selection less clear. The second and most important point is the cumbersome post-processing. This method requires manual selection of the frame at which each TS occurs, which is a time consuming subjective task that relies on the decision making of the individual selecting the frame.

Several of the methods using IMU data to detect TS are based on the traditional modelling of the skier movement as an inverted pendulum [7,17,46]. This modelling is based on the assumption that the neutral position of the pendulum occurs during the straight skiing moment between consecutive turns. Consequently, it would correspond with the instant of maximum angular velocity in the roll angle (Martinez CTD [43]), of zero lateral acceleration (Yamagiwa IMU [27]), of zero angular velocity in the yaw angle (Martinez Yaw CTD), and of zero angle with respect to the vertical (Yu IMU [28]). Among the methodologies based on the inverted pendulum concept, Martinez CTD and Yamagiwa IMU performed best. They showed an average accuracy between long and short turns within 0.01 s and 0.05 s respectively, and similar values independent of turn size. However, the precision for short turns is much smaller in both cases, potentially indicating better performance with more dynamic movements. It should be noted that these two methods do not require calibration, drift corrections, or other processing steps. They use the raw data, which would facilitate their potential use in trackers or other user-friendly applications. MCS was also used to assess Martinez CTD and Yamagiwa IMU’s performance. The IMU data was more accurate than MCS in both methods and had a similar precision for Yamawiga IMU, yet slightly worse for Martinez CTD. The reason for this difference could be that the placement and orientation of the IMUs is not totally aligned with the segment re-creation and orientation performed to the 3D data to match the signals. This could contribute to the statistically significant differences found with pairwise comparisons between the systems for both methods. However, the high number of data points could lead to a type 1 error, where negligible differences are deemed significant (see Table 2). Nonetheless, the system comparison supports the good performance of IMUs to detect TS using these methods. Another method based on the inverted pendulum was proposed by Yu et al. [28]. They counted turns (therefore selecting TS) based on the computation of the pelvis angle while skiing, which was measured from a single IMU. In the current study, the performance for the short turns, especially the precision, was considerably better than for long turns. This could be caused by the inherent drift when integrating the signal. Although a drift correction was performed, any remaining drift or signal noise would accumulate and amplify the divergence between the detected TS and the reference.

The combination of the signals from several IMUs allows for more complex post-processing, and in some cases for the reproduction of a whole body model. Three of the methods compared in this study were based on such computations. Fasel Ank CoM IMU [15] and Ulrich IMU [13] used the total distance and vertical distance between the CoM and the ankle joint center, respectively. Alternatively, Gerber et al. [14] based their method on the knee abduction-adduction angles (Gerber IMU). When compared to MCS, these three methods showed statistically significant differences, and in the first two cases, with over 0.14 s difference between the medians. The methods based on the IMU body model applied in this study did not reflect good results. The performance of these methodologies will be highly dependent on the accuracy of the reproduced body model. In the current study, the procedures to collect the data, calibrate the IMUs, and process the data replicated the original methodology previously validated. Consequently, this difference in the results between MCS and IMUs must come from the performance of the calibration movements, which is the basis for the IMU orientation and the resulting segment placement in space. This exemplifies the complexity and expertise needed to apply those methods. Nevertheless, none of these methods (with either system) performed better than the previously discussed methods based on simpler IMU configurations and processing. Consequently, because the individual IMU signals would already be included in a configuration aiming to reproduce a body model, the results indicate that the simpler methods would be most efficient.

It has been well established that the pressure measured with PI is not equal to the force transmitted to the skis during skiing [34,35]. While the initial intent was to assess kinetic methodologies using both PI and PFP, due to the weight of the system, only two participants were able to ski using the PFP system [33]. Consequently, the performance interpretation of the kinetic methods was made from the pressure data. Nakazato [34] and Spörri [36] based their methods on the detection of the point of minimum vertical load. In the current study, both methods showed a very good performance, with their accuracy being within 0.03 s and 0.01 s, respectively. In these methods, turn length and dynamics seemed not to influence the detection of TS, which is something to consider when selecting the most adequate TS detection method. The only difference between both methods is that while Nakazato directly selected the instant of minimum vertical load as TS point, Spörri approximated what would be the point of minimum vertical load based on the pressure dynamics for each turn. This methodological difference seemed to slightly enhance the accuracy of the Spörri TS detection. The results of the current study agreed with the results already reported by Martinez et al. [5]. The authors concluded that the methodology that defined TS as the point of equal pressure between the right and the left foot (Martinez Crossing) was detecting a different (earlier) event than the minimum vertical load. In the current comparison, the point of equal pressure also occurs consistently before the reference TS. The last method using kinetic data proposed by Vaverka et al. [37] defined a threshold based on the skier’s mass and inclination of the slope. This threshold was crossed at approximately 0.2 s before the reference TS for short turns and it was never crossed during long turns based on PI data. However, this method is based on the force measured with instrumented bindings and the pressure measured using PI is generally much lower than the force applied to the ski [34,35]. Further studies using force measuring devices in the bindings or skis with less cumbersome equipment would be needed to evaluate this method. Nonetheless, Spörri PI and Nakazato PI represent valid options to detect TS kinetically due to the ease of use of the PIs and their performance.

Kröll et al. [32] proposed a method based on raw EMG data and the knee flexion angle (Kröll EMG). Although the identified time point occurs consistently 0.2 s before the reference TS point, it is very precise (≈ 0.10 s). The excellent precision of this method along with the early detection compared to the reference seems to indicate that this method identifies a characteristic muscle activation needed to produce a TS movement. It seems clear that with awareness of the consistent offset between the event detected and the reference, this methodology would be a valid option to segment turns in datasets based on EMG signal.

Several of the methods evaluated presented better values for accuracy and precision during short turns compared with long turns. Although the ski treadmill was intended to replicate the typical movement of skiing, the turning dynamics were limited by the specifics of the treadmill. Both turning styles were performed at the same speed. Consequently, since long turns on snow would typically be faster than short turns, the relative intensity and the dynamic nature of the short turns were higher and likely have more resemblance to on snow skiing. This could indicate that the methods with considerably better performance for short turns would most likely reflect the same (if not better) accuracy and precision during on snow skiing. However, it is also possible that they would be more affected by differences in technique and skill level between skiers on snow.

In summary, the selection of a TS detection methodology can be adapted to different requirements, such as sensor type, turn style, and ease of use. Yamagiwa [27] and Martinez CTD [43] would be the best methods when using IMUs, and the specific method selection could be based on the sensor placement or the type of IMU available. Spörri [36] and Nakazato [34] perform best among the kinetic methods. If only position data is available, the method proposed by Kondo et al. [31] seems to yield acceptably accurate results. However, if the necessary data to implement any of the aforementioned methods is available, choosing an IMU or PI based method would yield a more accurate detection than Kondo. Nevertheless, the one method that performed best overall was Klous [8] video frame selection of edge change. Although it is the most accurate and precise method, the possibility to automatize this method could influence the potential applications, and time required for data processing after collection. Between the aforementioned best performing methods, all have the potential to be automated with the exception of Klous [8]. Whether a method can be implemented in real-time or quasi real-time could also affect the potential future applications. The data processing steps needed for the methods Nakazato, Yamawiga, and Martinez CTD would be feasible to perform in real-time, or with a lag of a few turns. Alternatively, the method proposed by Spörri could be implemented after each ski run. From a practical standpoint, proper turn segmentation is the basis for the assessment of turn-by-turn ski metrics that can help to better understand skiing performance. This work showed the possibility to accurately detect turn switches with different sensors depending on the specific needs or equipment available. Ultimately, the specific TS methodology chosen for future applications should be carefully considered with respect to sensor selection, performance (accuracy and precision), and intended purpose.

## Figures and Tables

**Table 1:** Turn switch detection methodologies assessed. A brief explanation of the concept in the “Method” column. The abbreviation used to refer to each method in this manuscript is in the “Name” column. The number of participants included in each method and sensor for short and long turns is under “S [n]” and “L [n]” respectively.

Method	Name	Sensor Type	S [n]	L [n]
Crossing point between the CoM trajectory and the arithmetic mean of the skis’ trajectories [3]	Supej MCS *	Motion capture	14	13
	Supej IMU	Inertial meas. unit	1	3
Ski edge change frame selection [8]	Klous Video	Video	13	11
Minimum value of the ground reaction force/ pressure [34]	Nakazato PI	Pressure insole	11	7
	Nakazato PFP	Force platform	2	2
Combination of knee angle and pronounced EMG amplitude decrease [32]	Kröll EMG	Motion capture + muscle activity	9	10
Point when resultant reaction force equals the force acting perpendicular to the ground [37]	Vaverka PI	Pressure insole	10	-
Inflection point in the head trajectory [31]	Kondo MCS	Motion capture	14	13
Zero crossing in the lateral axis of an accelerometer placed on the upper torso [27]	Yamawiga MCS	Motion capture	14	12
	Yamawiga IMU	Inertial meas. unit	12	11
Change in sign of the angular velocity component calculated from position data [30]	Adelsberger MCS	Motion capture	13	12
Functional minima of the ground reaction force/ pressure [36]	Spörri PI	Pressure insole	11	7
	Spörri PFP	Force platform	2	2
Zero crossing of the pelvis roll angle [28]	Yu IMU	Inertial meas. unit	9	8
Intersection between right and left knee abduction-adduction angle [14]	Gerber MCS	Motion capture	14	12
	Gerber IMU	Inertial meas. unit	5	4
Intersection of right and left vertical distance between CoM and ankle joint center [13]	Ulrich MCS	Motion capture	14	12
	Ulrich IMU	Inertial meas. unit	9	7
Intersection of right and left total distance between CoM and ankle joint center [15]	Fasel Ank CoM MCS	Motion capture	13	10
	Fasel Ank CoM IMU	Inertial meas. unit	6	7
Inflection point of the CoM trajectory [15]	Fasel CoM IP MCS	Motion capture	14	12
Peak angular velocity in the roll axis of the shank [43]	Martinez CTD	Instrumented boot	10	8
	Martinez MCS	Motion capture	14	10
Crossing between right and left vertical ground reaction force [5]	Martinez Crossing PI	Pressure insole	9	9
	Martinez Crossing PFP	Force platform	1	1
Zero crossing of the average right and left shank angular velocity in the yaw axis [new]	Martinez Yaw CTD	Instrumented boot	10	7
	Martinez Yaw MCS	Motion capture	14	12

* Reference methodology; ST, short turns; LT, long turns; CoM, center of mass; MCS, motion capture system; IMU, inertial measurement unit; PI, pressure insoles; PFP, portable force plates; EMG, electromyography; Ank, ankle; IP, inflexion point; CTD, instrumented boot; n, size of the sample.

**Table 2:** List of methods assessed with two different sensors, the absolute median difference between both sensors and all the turns pooled together, and Wilcoxon signed ranks test statistic.

Method	Sensors	Diff [s]	*p*-Value	n
Nakazato	PFP vs. PI	0.04	0.755	15
Yamagiwa	MCS vs. IMU	0.14	<0.001	330
Spörri	PFP vs. PI	0.00	0.140	15
Gerber	MCS vs. IMU	0.18	<0.001	135
Ulrich	MCS vs. IMU	0.15	<0.001	225
Fasel Ank CoM	MCS vs. IMU	0.01	<0.001	176
Martinez	MCS vs. IMU	0.04	<0.001	239
Martinez Crossing	PFP vs. PI	0.05	0.045	28
Martinez Yaw	MCS vs. IMU	0.14	<0.001	255

CoM, center of mass; MCS, motion capture system; IMU, inertial measurement unit; PI, pressure insoles; PFP, portable force plates; Ank, ankle; diff, difference; ms, milliseconds; n, size of the sample (all pooled turns).

## Data Availability

The data presented in this study are available on request from the corresponding author.
